# Notes and News

**Published:** 1903-12-12

**Authors:** 


### NOTES AND NEWS.

The King has presented a framed photograph of himself to the Princess Alice Hospital, Eastbourne, and he has sent a present of game to St. Peter's Hospital, Henrietta Street, and of cast linen to St. George's Hospital.

The new wing at the Huddersfield Infirmary is the gift of Colonel E. H. Carlile, J.P., who has defrayed the whole cost of building and furnishing at an expenditure of £20,000.

The new buildings of St. John's Hospital, Twickenham, were opened recently by Mrs. Cunard. Mr. and Mrs. Cunard have supported the hospital liberally since its foundation, and with their assistance the cost of the present additions have been defrayed.

The John Fowler Memorial Sanatorium for Children, at Liverpool. The property adapted for the purpose has been purchased by Miss Fowler, of Sefton Park, who has devoted £20,000 to the object of securing a hospital and home for delicate children. The Institution will form part of the organisation founded by Dr. Stephenson, and known as the Children's Home, Bonner Road, London.

An outbreak of diphtheria has occurred at the Chester Park Schools, Bristol. The question of the medical inspection of school children was brought up in connection with this at the last meeting of the Bristol Health Committee, and it was pointed out that as the Health Committee and the School Board were both Committees of the City Council and that former difficulties in making such arrangements should not now be experienced. There can be no question that any means which tended to check an epidemic must prove a saving to the ratepayer.

There are now 124 cases of plague under treatment at Rio de Janeiro.

A new isolation hospital has been erected for Maldon at Heybridge, at a cost of £5,600.

The late Mr. Attwood-Matthews, of Hereford, has bequeathed £5,000 to the Herefordshire General Hospital.

A new operating theatre and boardroom have been opened at the Carnarvonshire and Anglesey Infirmary.

Upwards of 255,000 rats have been destroyed at the Port of London since the work of extermination was commenced.

The Plymouth Dental Hospital has opened new and improved premises in Regent Street, which provide an operating-room with efficient dental apparatus.

On Friday, at three o'clock, Princess Victoria of Schleswig-Holstein has convened a drawing-room meeting at Schomberg House, Pall Mall, on behalf of the East Berkshire Branch of The League of Mercy, of which she is lady president.

The late Mr. Aaron Abecasis, of Maida Vale, has bequeathed £1,000 to the London Hospital to found a bed in the Jewish ward, and £500 to the Great Ormond Street Hospital for Children, to found a cot, and the ultimate residue of his property in trust to such hospitals having wards for the reception of Jewish patients as his executors shall select.

The will of the late Sir John Blundell Maple provides that his executors shall provide for the rebuilding of University College Hospital to the extent of £200,000. He expresses a wish that Lady Maple shall devote £3,000 to charitable purposes within the Metropolitan area and St. Albans and Harpenden; £20,000 is also to be utilised for the same purpose at Lady Maple's death.

The new building of the North-Eastern Hospital for Children, Hackney Road, has now been completed, and the hospital is in consequence working with 114 beds instead of 57 as formerly. The official opening ceremony is postponed to next year, but meanwhile the new wards are being used to the great benefit of the poor children in the crowded district of Bethnal Green. The cost of maintenance will necessarily be much increased, seeing that the number of beds has been doubled, and at least £3,000 a year will have to be added to its income. The need for increased support is now very great. There is a debt of £10,000 on the new building, while £25,000 must be raised to complete the scheme of enlargement which has been adopted with the view of raising this hospital to the modern standard of efficiency. Mr. T. Glenton-Kerr, the secretary, will gratefully acknowledge contributions.

